# Perspectives for restriction of dental treatment complexity during and after Covid-19

**DOI:** 10.1186/s42269-022-00739-7

**Published:** 2022-03-03

**Authors:** Sadaf Sadat Mahmoudinezhad, Kooshan Moradi, Neda Boushehri

**Affiliations:** 1 Department of Periodontics, School of Dentistry, Ahvaz Jundishapur University of Medical Sciences, Ahvaz, Iran; 2 Department of Pediatrics Dentistry, School of Dentistry, Babol University of Medical Sciences, Babol, Iran

To the editor,

## Background

In early 2020, a novel β-coronavirus called SARS-CoV-2 caused a severe acute respiratory syndrome (coronavirus disease 2019 (COVID-19)). It has rapidly spread throughout the world, and on March 11, 2020, the WHO declared COVID-19 as a pandemic (Guo et al. 2020). As of November 12, 2021, there have been more than 252,113,814 confirmed cases of COVID-19 in 192 countries around the world, and more than 5,083,105 people have died due to this disease (Coronavirus Resource Center 2021).

It spreads through the respiratory tract by droplets, respiratory secretions, and direct contact. The incubation period ranges from 1 to 14 days, with an average of 3–7 days (Guo et al. 2020). Although contact with symptomatic patients may be a route of transmission, asymptomatic individuals or those within the viral incubation period may also be threatening for others (Kampf et al. 2020). All dental procedures may produce aerosols that fall onto a surface that the dentist, dental assistant, and even patients may later contact. Respiratory droplets contaminating surfaces and proximity of the dentist to the patient's oropharyngeal region who may have COVID-19 disease may put the dentist, employers, and other patients at the dental office at the risk of cross-contamination (Ather et al. 2020). Therefore, it is very common that dentists and patients develop a fear of being infected at the dental office.

## Main text

Since the beginning of the pandemic, changes in dental care-seeking behavior, restrictions for elective dental treatments, and monetary problems caused by the pandemic have increased dental infections, maxillofacial pain, and more complicated dental treatments. Vaccination of people seems a favorable preventive strategy for reducing the impact of COVID-19 on oral hygiene of people these days. It is also essential to vaccinate most of the population soon in developing countries and countries with large populations with restricted access to vaccination. Besides, the appearance of recent variants of COVID-19 may threaten the control of the pandemic, especially in developing countries. The efficacy of current vaccines on new strains is also questionable. Therefore, coherent planning for the rehabilitation of public oral health, including prevention and elective and emergency dental treatment, is crucial during and after the pandemic.

The first step to manage the outcomes mentioned above is to consider tele-screening and triaging to identify suspicious patients remotely when scheduling appointments (Mahmoudinezhad et al. 2020). Additionally, careful prescreening like asking patient's travel history and measuring temperature using a non-contact forehead thermometer, wearing a surgical mask, covering the mouth and nose with a tissue while coughing, disinfecting inanimate public contact consistently and correctly with various types of hospital-level disinfectants (such as sodium hypochlorite and 70% ethanol) are the other ways to make the dental office a safer place for treating the emergency dental condition. Asking patients to rinse their mouth with hydrogen peroxide 1% or 0.2% and povidone-iodine mouthwash prior to their dental treatment might reduce the load of coronaviruses in saliva (Kariwa et al. 2006; Dorestan et al. 2021). Using a rubber dam, high volume suction, extra-oral imaging, minimizing the use of ultrasonic instruments, and high-speed handpieces could reduce the risk of generating contaminated aerosols (Ather et al. 2020).

Oral diseases are a costly economic burden for individuals, families, and nations, both industrialized and developing. Consequently, it should be additional governmental support (insurance and finances) for reducing the economic pressure on patients for their dental treatments to reduce the impact of COVID-19 on public oral health. They should also provide more accessible dental health centers to facilitate patients' referrals, as well.

The final solution for having a balanced healthcare outcome is to have some educational interventions for people about how to detect and prevent tooth caries' progression. For effective oral health promotion, oral health instructions should be spread among the high percentage of people in the society by social media, well-designed brochures, face-to-face oral instructions, demonstration of oral hygiene practices at the dental clinic or provincial health centers, and asking dental students for volunteering actions under particular infection prevention protocols for COVID-19. Also, developing oral health education applications and video games and expanding their use in the community can help to improve oral health.

## Conclusions

In conclusion, tele-dentistry, new technologies such as new applications and games designed for oral health education, and social medias can reduce the risk of dental caries and increase the early detection of dental caries and any other oral diseases. In addition, we suggest that dentists and software engineers have more cooperation for more integration of different aspects of the dentistry, such as oral health education and public awareness of oral diseases, with the world of technology, especially during the pandemic period.

## Data Availability

The corresponding author had full access to all the data in the study and take responsibility for the integrity of the data and the accuracy of the data analysis.
